# Insight into the Effect of Counterions on the Chromatic Properties of Cr-Doped Rutile TiO_2_-Based Pigments

**DOI:** 10.3390/ma15062049

**Published:** 2022-03-10

**Authors:** Xiaojian Zhou, Xiaozhen Zhang, Chunhai Zou, Renhua Chen, Lanlan Cheng, Botao Han, Huafeng Liu

**Affiliations:** 1 School of Materials Science and Engineering, Jingdezhen Ceramics University, Jingdezhen 333403, China; zhouxiaojian157@163.com (X.Z.); boseahan@163.com (B.H.); 2 Jiangxi Jinhuan Pigments Co., Ltd., Yichun 336000, China; chenrenhua1979@126.com (R.C.); chenglanlan151@126.com (L.C.); 3 Shenzhen Customs Logistics Management Center, Shenzhen 518067, China; zouch66@163.com

**Keywords:** inorganic pigments, rutile titania, counterion, chromatic performance, spectroscopic properties, chemical stability

## Abstract

Rutile TiO_2_ pigments codoped with chromophore ion Cr^3+^ and various charge-balancing ions (i.e., counterions species of Sb, Nb, W and Mo) were prepared by a solid-phase reaction method. The effects of the counterions and calcination temperatures on the phase structure, color-rendering and spectroscopic properties, microstructure, and stability of the synthesized pigments were investigated in detail. The results showed that the introduction of 5–10% counterions improved the solubility of Cr^3+^ in the TiO_2_ lattice to form the single-phase rutile pigments calcined at 1100 °C for 2 h. The 10% Cr-doped pigment showed a dark brown color. Depending on the content and type of counterions, the color of the codoped pigments was tailored from yellow to reddish or yellowish-orange to black with different brightness and hue. The influence mechanism of counterions was ascribed to the lattice distortion and variation in the charge balance condition. It was found that the addition of Sb, Nb, or Mo resulted in a remarkable improvement in the NIR reflectance of pigments. The grain growth was inhibited with the codoping of Cr/Sb and Cr/Nb to achieve the nano-sized pigments. In addition, the prepared pigments exhibited good acid and alkali corrosion resistance as well as excellent stability and coloring performance in transparent ceramic glazes.

## 1. Introduction

Inorganic pigments are widely used as important colorants for various applications such as coatings, glasses, ceramics, plastics, paints, inks, enamels, and construction materials due to their outstanding thermal stability and high chemical stability as well as excellent coloring ability compared to organic counterparts [1,2,3,4,5,6]. In recent years, with the strong regulation of environmental protection departments and increasing concerns regarding environmental issues, the demand for environmentally friendly non-toxic pigments has increased. However, most of today’s brightly colored and widely available inorganic pigments contain toxic heavy metal elements as the main component (e.g., CdS/CdSe, PbCrO_4_, Sb_2_O_5_·2PbO) and their applications are severely restricted [7]. Therefore, the development of eco-friendly and cost-effective inorganic pigments has become a popular research topic in the area of pigments [8,9,10].

Titania (TiO_2_) is an abundant and cheap compound of polymorphic forms including anatase, rutile, and brookite. Among them, rutile TiO_2_ has a tetragonal crystal structure composed of the [TiO_6_] octahedra, which are connected to each other by ribs to form a relatively stable chain extending along the c-axis, and the chains are linked by the common corner tops of the octahedra [11,12]. Therefore, the rutile TiO_2_ possesses a thermodynamically stable crystalline structure, which makes it an excellent material of oxide pigments with high chemical and thermal stability. Furthermore, the high refractive index of rutile TiO_2_ endows the pigments with strong opacifying ability. However, the pure rutile TiO_2_ can only be used as the white pigment without visible-light absorption due to a wide bandgap of about 3.0 eV, which would greatly limit its decorative effects and thus industrial applications [13,14,15,16]. Therefore, the elemental doping modification strategy was applied to adjust the bandgap and spectral properties of rutile TiO_2_ for developing pigments with different colors [17,18,19]. Currently, several elements as chromophore ions with valence states below 4+ such as Cr, Ni, Mn, Fe, Co or V were doped into the rutile lattice to substitute for Ti^4+^, obtaining pigments with different colors from orange to yellow to brown to gray, and even to green [20,21,22]. In order to balance the charge difference between the chromophore (such as Cr^3+^) and Ti^4+^, a second ion with higher valence states (so-called counterion, e.g., Sb, Nb, W and Mo) needs to be introduced to achieve the desired chromatic properties [19,23,24]. It was found that the coupling of chromophore ions and counterions has significant influences on the anatase to rutile transition of TiO_2_, the spectral properties, and thus the color-rendering performance, depending on the type of ion pairs and doping contents. In particular, the doping of chromophore ions proved to be dominant in reducing the onset temperature of the anatase to rutile transformation by 50–300 °C [12,22,25,26].

The Cr-doped TiO_2_ is one of the most-researched rutile pigments, in which Sb^5+^ as a counterion was commonly introduced to attain the orangish-yellow color [17,27,28]. The doped Sb ions in a small amount are incorporated into the stable rutile lattice to form a solid solution, and cannot be easily leached out in applications. Therefore, the Sb-doped rutile pigments are generally considered eco-friendly compared to the above-mentioned toxic pigments with a high content of heavy metal and relatively bad stability. The Cr-doped rutile pigments also exhibited good chemical and thermal stability as well as high near-infrared radiation (NIR) reflectance, which is desired for colored cool pigments. As shown by many researchers [28,29,30,31,32], the codoping of counterions is an effective means to tailor the color of TiO_2_-based rutile pigments for various decoration applications. However, few studies have systematically investigated the role of counterions with different contents and valences on the phase structure and chromatic performance of the rutile TiO_2_ pigments. Therefore, in this work, different counterion species (Sb, Nb, W, or Mo) were codoped with chromophore ion Cr^3+^ to prepare the rutile TiO_2_-based pigments by a low-cost solid-state reaction method. We systematically investigated the effects of counterion types and doping amounts (5% and 10% in molar ratio) on the phase composition, color-rendering and spectroscopic properties, and morphology of the prepared TiO_2_-based rutile pigments. In addition, the stability of the synthesized pigments in terms of acid and alkali resistance and their application properties in ceramic glazes were further evaluated. The present research aims to develop eco-friendly pigments for energy-saving building decorative paints, plastic coloring, and ceramic decoration.

## 2. Experimental Procedure

### 2.1. Materials

Metatitanic acid (TiO(OH)_2_), molybdenum trioxide (MoO_3_), tungsten trioxide (WO_3_), and niobium pentoxide (Nb_2_O_5_) were purchased from Shanghai Aladdin Technology Co., Ltd, Shanghai, China. Chromium trioxide (Cr_2_O_3_), antimony trioxide (Sb_2_O_3_), and anhydrous ethanol (C_2_H_5_OH) were supplied by Sinopharm Chemical Reagent Co. Ltd, Shanghai, China. All raw materials were of analytical purity grade and used without further purification.

### 2.2. Synthesis of Ti_0.9−x_Cr_0.1_M_x_O_2_ Pigment

The TiO_2_-based pigments with the general formula Ti_0.9−*x*_Cr_0.1_M*_x_*O_2_ (where M = Sb, Nb, Mo, and W; *x* = 0, 0.05 and 0.10) were synthesized by a low-cost solid-state reaction route. All chemical reagents with a designed stoichiometric ratio were mixed by ball milling with toughened zirconia balls for 3 h at 450 rpm in ethanol to obtain a homogeneous slurry. The slurry was dried in an oven at 75 °C. The dried mixture was then transferred to an alumina crucible and calcined at 950–1100 °C for 2 h in air at a heating rate of 3 °C/min. To evaluate the coloring performance and stability of prepared pigments in ceramic glazes, Ti_0.9−*x*_Cr_0.1_M*_x_*O_2_ was mixed with a transparent glaze powder in a mass ratio of 5%, and then fully dispersed in an appropriate amount of water to obtain a homogeneous glaze slurry. The slurry was coated on the ceramic green bodies and heated up to 1000 °C for 20 min to obtain the samples of colored glaze. The chemical composition of the used transparent glaze powder is shown in Table 1.

### 2.3. Characterization Techniques

The crystalline structure of the pigment powders was characterized by X-ray diffraction (XRD) using Cu-Kα (λ = 0.15418 nm) radiation with a D8 Advance diffractometer operated at 40 kV for 30 mA. The XRD data were collected from a 5 to 80° 2θ range with a step size of 0.02°. The pigment precursors were characterized using a simultaneous TG-DSC thermal analyzer (STA449C, Netzsch-Gertebau GmbH, Selb, Germany), heating from room temperature at a rate of 10 °C/min to 1100 °C. The UV–Vis diffuse reflectance spectra (200–900 nm) and near-infrared reflectance spectra (780–2500 nm) of the pigment samples were measured by a UV–Vis–NIR spectrophotometer (Lambda 950, Perkin-Elmer, New York, NY, USA). BaSO_4_ was used as the reference substance, and the scanning interval was 2 nm. The bandgap of the sample was calculated according to the UV–Visible spectrum. The specific calculation process is as follows: according to the UV–Visible reflectance R of the samples, E = 1240/λ is used as the horizontal coordinate. F(r) is calculated by the Kubelka–Munk formula: F(r) = (1 − R)^2^/2R, and (F(r)*E)^2^ is used as the vertical coordinate. The graph is extrapolated as a tangent, and the intercept of the tangent on the horizontal axis is the bandgap (E_g_) [21,33,34]. The colorimetric parameters of the pigments and colored glaze in the CIELab system were measured using an Automatic Whiteness meter (WSD-3C, Beijing, China), in which L* is the Lightness axis (black (0)/white (100)), a* is the green (−)/red (+) axis and b* is the blue (−)/yellow (+) axis. The color saturation C* is defined as C* = [(a*)^2^ + (b*)^2^]^1/2^. The microstructure of the samples was analyzed by a field emission scanning electron microscopy (FE-SEM, SU-8010, Hitachi, Tokyo, Japan) operated at 5 kV. To evaluate the chemical stability of the pigments, the samples synthesized by calcination at 1100 °C for 2 h were soaked in 5 wt% H_2_SO_4_, HCl, and NaOH aqueous solution for 60 min, respectively. After washing and drying, the acid and alkali corrosion resistance of samples was evaluated by comparing the color difference before and after treatment.

## 3. Results and Discussion

**Figure 1:**
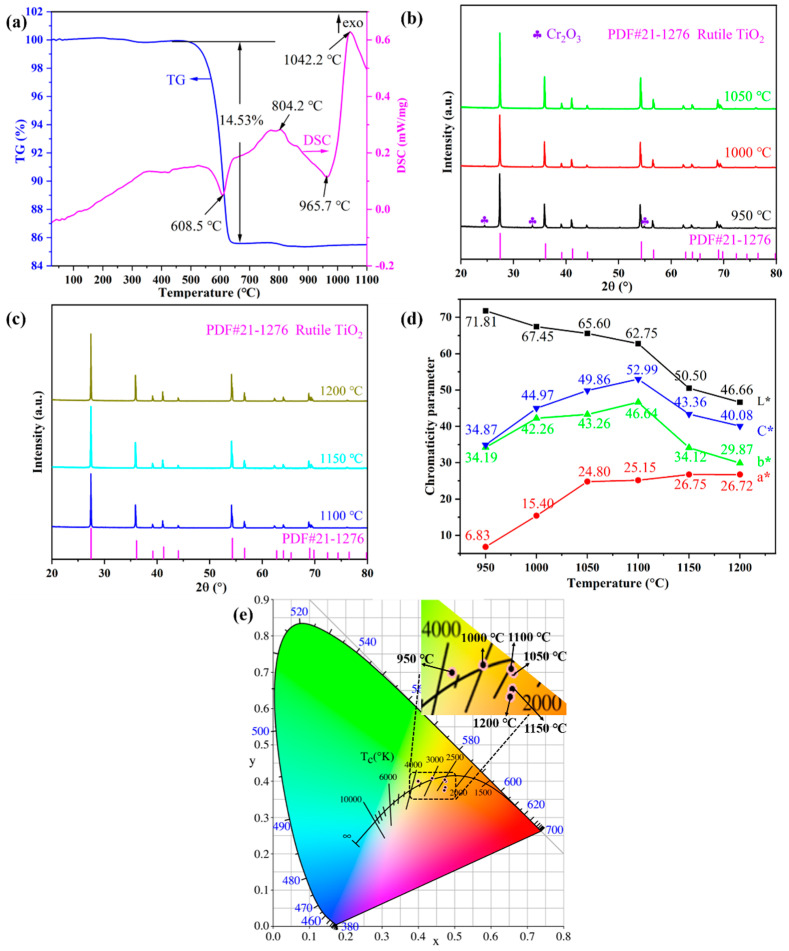
TG–DSC curves of Ti_0.8_Cr_0.1_Sb_0.1_O_2_ precursors (**a**); powder XRD patterns synthesized at different temperatures (**b**,**c**), CIELab chromaticity parameters (**d**), and chromaticity coordinates (**e**) of the as-synthesized pigments at different temperatures.

The combined DSC-TG thermal analysis and XRD measurements were conducted to study the physical and chemical changes of the pigment precursor during the heat treatment. Figure 1a shows the DSC–TG curves of the Ti_0.8_Cr_0.1_Sb_0.1_O_2_ pigment precursors heated up from room temperature to 1100 °C. The total mass loss was about 15%, of which the vast majority (14.5%) occurred at around 600 °C, as shown in the TG curve. Accordingly, a sharp endothermic peak appeared near 608 °C in the DSC curve, which was mainly caused by the decomposition and dehydration of metatitanic acid (hydrated TiO_2_). Exothermic peaks also appeared in the DSC curve near 804 °C and 1042 °C, but no noticeable mass loss could be observed in the corresponding TG curve. Therefore, the two exothermic peaks were caused by the generation of the anatase TiO_2_ crystal phase and the phase transformation of anatase to rutile, respectively, which was similar to the reported results in the literature [4]. The transformation process of the anatase to rutile phase is exothermic, and the transition temperature generally ranges from 400 to 1200 °C, depending on the raw material properties, doped ions, synthesis method, and heat treatment system, etc. [25]. Figure 1b,c present the XRD patterns of Ti_0.8_Cr_0.1_Sb_0.1_O_2_ pigments calcined at 950–1200 °C for 2 h. It can be seen that the powder calcined at 950 °C showed the main crystalline phase of rutile TiO_2_ (PDF#: 21-1276) while a trace Cr_2_O_3_ phase also existed, suggesting that the solid-phase reaction was not fully completed. When the temperature was increased up to 1000–1200 °C, only a single rutile TiO_2_ phase was obtained without any impurity, which indicates that Cr^3+^ and Sb^5+^ cations completely dissolved in the TiO_2_ lattice [23]. Note that the introduced Sb^3+^ by Sb_2_O_3_ would be oxidized to Sb^5+^ in the calcination process [4,35,36]. The XRD results showed that the temperature for the formation of the rutile phase was lower than the exothermic peak temperature (about 1042 °C) generated by the phase transformation of anatase to rutile, as shown Figure 1a. This may be due to the temperature lag caused by the rapid temperature rise during the thermal analysis. In addition, the intensity of the diffraction peaks increased gradually with increasing temperature, indicating that the crystallization of the synthesized pigment was promoted. Combined with the color-rendering properties shown in Figure 1d, the optimal calcination temperature can be determined as 1100 °C, at which a bright yellow Ti_0.8_Cr_0.1_Sb_0.1_O_2_ pigment with the highest yellowness value (b*) and thus more vivid color can be synthesized.

Figure 1d shows the CIELab chromaticity parameters of Ti_0.8_Cr_0.1_Sb_0.1_O_2_ pigments calcined at 950–1200 °C for 2 h. It can be seen that the calcination temperature had an obvious influence on the chromatic performance of Ti_0.8_Cr_0.1_Sb_0.1_O_2_ pigments. With the increase in temperature, the L* value (lightness) presented a decreasing trend. In particular, when the temperature was ≥1150 °C, a significant decrease in the L* value could be observed, which might be due to the reduction in Ti^4+^ to a lower valence at higher temperatures, generating free electrons and consequently resulting in enhanced absorption of visible light [37]. The a* value (redness) of pigments increased with increasing temperature, while the b* value (yellowness) increased first and then decreased. At 1100 °C, the highest b* value of 46.64 was achieved due to the full incorporation of Cr^3+^ and Sb^5+^ into the TiO_2_ lattice, which was much higher than those (b* < 40) of the Cr/Sb codoped pigments synthesized via the precipitation or hydrothermal methods [23]. Accordingly, the obtained pigment exhibited the highest C* value (color saturation) of 52.99. The chromaticity coordinates of the samples calcined at different temperatures are shown in Figure 1e, which are consistent with the results in Figure 1d. The above results show that the doped TiO_2_-based pigment synthesized at 1100 °C for 2 h possessed the best chromogenic properties with high yellowness value and color saturation.

**Figure 2:**
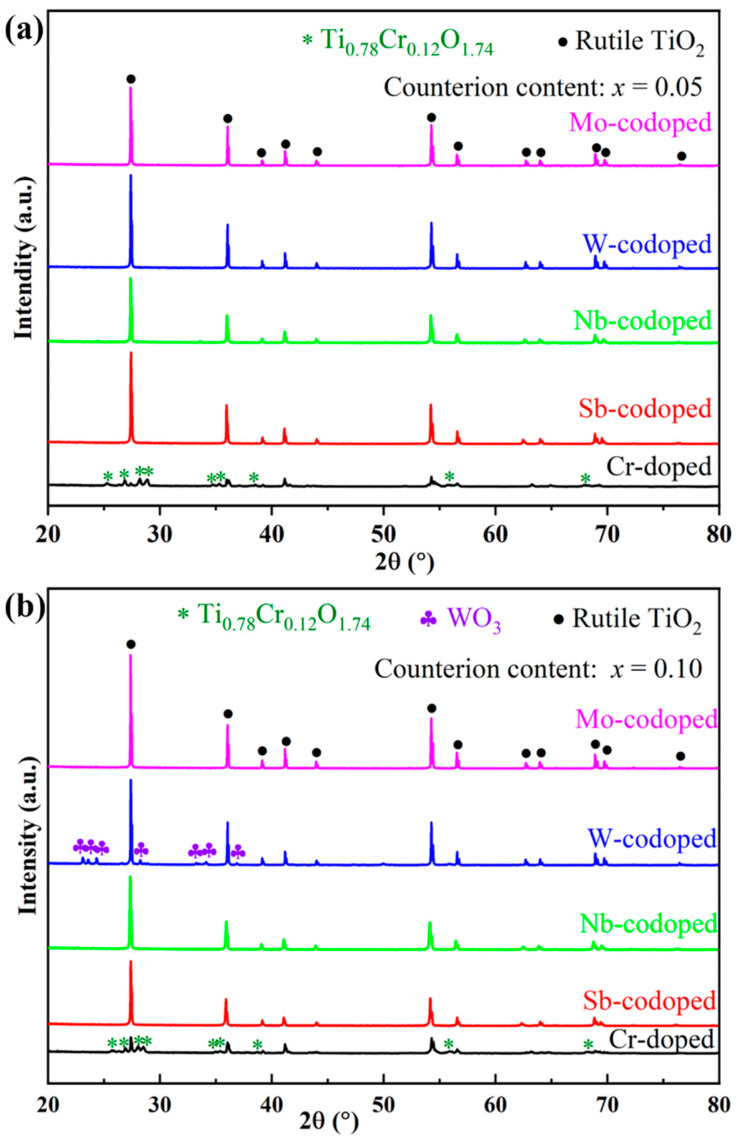
XRD patterns of Ti_0.9_Cr_0.1_O_2_ and Ti_0.9−*x*_Cr_0.1_M_*x*_O_2_ (M = Sb, Nb, W, Mo) pigments at different counterion contents: (**a**) *x* = 0.05 and (**b**) *x* = 0.10.

To investigate the effect of different high-valence counterions on the Cr-doped TiO_2_-based pigments, Ti_0.9−*x*_Cr_0.1_M*_x_*O_2_ (M = Sb, Nb, Mo and W; *x* = 0, 0.05 and 0.10) powders were synthesized at 1100 °C for 2 h and characterized in terms of phase composition, chromatic performance, and spectroscopies. Figure 2 shows the XRD patterns of obtained pigments doped with different counterions at 5% and 10% (in molar ratio). As can be seen from Figure 2a, for the sample with a nominal composition of Ti_0.9_Cr_0.1_O_2_, an impurity of chromium–titanium oxide (Ti_0.78_Cr_0.12_O_1.74_) was formed in addition to the rutile phase. With the codoping of 5% counterions (Sb, Nb, W, or Mo ions), all the characteristic diffraction peaks of the obtained pigment powders could be well indexed to the rutile TiO_2_ structure (PDF#21-1276) [20], which indicates that both the counterions and Cr^3+^ had entirely incorporated into the TiO_2_ lattice to form a solid solution. The XRD analysis showed that the introduction of counterions could help to improve the solubility of Cr^3+^ in the rutile lattice, which may be due to, on one hand, lattice distortion caused by the radius difference of codoped ions improving the capability of the rutile structure to accommodate Cr^3+^; on the other hand, the counterions with higher valence could reduce the charge imbalance of TiO_2_ caused by lower valence Cr^3+^ and keep the whole system electrically neutral [38]. As shown in Figure 2b, when the doping level of counterions was increased to 10%, the Sb, Nb, or Mo-codoped Ti_0.9_Cr_0.1_O_2_ maintained a single rutile structure phase, while for the W-doped sample, a minor impurity of WO_3_ was also formed, indicating that the solubility of W in the TiO_2_ lattice was lower than that of other counterions. This phenomenon was similar to that of the Ti_0.9_Cr_0.1_O_2_ pigment, but the introduction of 10% high valent W^6+^ would lead to surplus positive charges, so not all of the W^6+^ could be incorporated into the rutile lattice.

**Figure 3:**
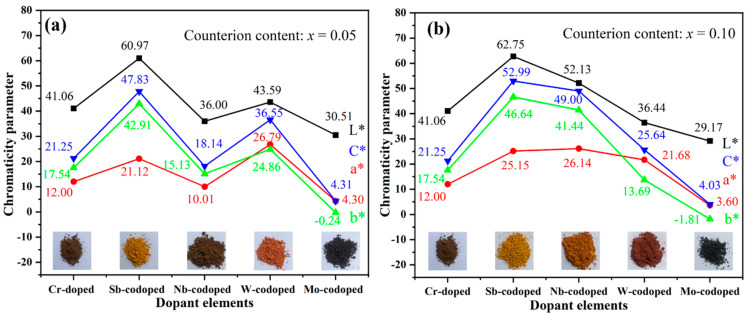
CIELab chromaticity parameters of Ti_0.9−*x*_Cr_0.1_M_*x*_O_2_ pigments codoped with different counterions and calcined at 1100 °C for 2 h: (**a**) *x* = 0.05; (**b**) *x* = 0.10.

**Figure 4:**
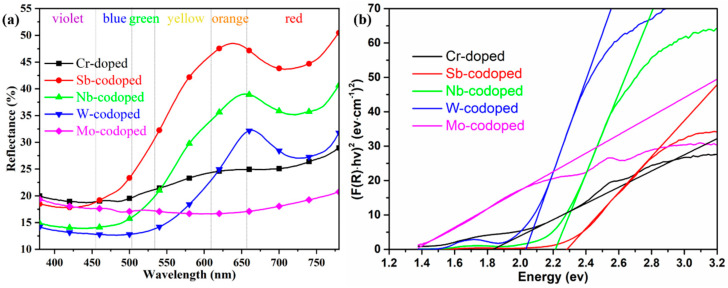
UV–Vis reflection spectra (**a**) and derived bandgap (**b**) of 10% Cr-doped TiO_2_ pigments with and without 10% counterions.

Figure 3 shows the values of CIELab chromaticity parameters of Ti_0.9−*x*_Cr_0.1_M*_x_*O_2_ (M = Sb, Nb, Mo, and W; *x* = 0, 0.05 and 0.10) pigments. As can be seen from Figure 3a, the Ti_0.9_Cr_0.1_O_2_ pigment had a dark brown color with low a* and b* values. After codoping with 5% Sb, W, or Mo, respectively, the color rendering performance of the obtained pigments varied greatly. Among them, the Sb-codoped pigment showed a typical yellow color with relatively high L* and b* values and low a* value, while the W-codoped one presented a reddish-orange with a reduced L* value and close a* and b* values. With the codoping of 5% Mo, a black pigment was obtained with the lowest L*, a*, and b* values. However, when codoped with Nb, the obtained pigment showed a dark brown color, which was similar to that of Ti_0.9_Cr_0.1_O_2_ without the codoping of counterions. As shown in Figure 3b, when the codoping amount of Sb was increased to 10%, a brighter yellow color could be achieved with obviously increased L*, a*, b* values and color saturation (C*). Surprisingly, for the Ti_0.8_Cr_0.1_Nb_0.1_O_2_ pigment, all the chromatic parameters enhanced markedly and a bright yellowish-orange color was obtained. However, the chromatic parameters of the Ti_0.9−*x*_Cr_0.1_W_*x*_O_2_ pigment decreased obviously with W content incrementing from 5% to 10%, and consequently the color darkened. The increase in Mo content had little effect on the color performance of the obtained pigment since only a minor decrease in L*, a*, b* parameters was observed in Figure 3. The variation of color rendering performance for the obtained rutile pigments can mostly be ascribed to the alteration of charge balance and degree of lattice distortion caused by the counterions with different valences, radii, and contents. For the 10% Sb^5+^ or Nb^5+^ codoped samples, the electric neutrality of crystal structure can be achieved, preventing the generation of electronic defects, and consequently, greatly enhanced yellowness (b* value) could be obtained. However, for the introduced counterion of Mo^6+^ by MoO_3_, it would be easily subjected to a reduction to lower valent Mo^3+/4+/5+^ [19,39,40], which would result in the occurrence of electronic defects and thus high visible light absorption rate, yielding a black-toned rutile pigment. Regarding the redox stable W^6+^, the electric neutrality was attained at the doping level of 5%, and thus the further increase in W^6+^ content to 10% led to the surplus positive charge and even residual second phase WO_3_, as shown in Figure 2b, resulting in greatly reduced chromatic parameters and darkening of the pigment. The influence of different counterions is further clarified below according to the UV–Visible spectroscopy results shown in Figure 4a. In conclusion, the obtained results show that multi-colored rutile TiO_2_-based pigments can be achieved by the rational design of incorporated counterions and their content to regulate the structure distortion of [TiO_6_] octahedrons and the degree of charge imbalance.

To further elucidate the effect of different counterions on the color-rendering properties of series Ti_0.8_Cr_0.1_M_0.1_O_2_ (M = Sb, Nb, W, Mo) pigments, the UV–Vis diffuse reflectance properties were also investigated, as shown in Figure 4a. It can be observed that the pigment doped with only 10% Cr showed a very low reflectance from yellow to orange to red bands with the wavelength in the range of 550–780 nm, which could well justify its dark brown color. The codoping of Sb and Nb resulted in a significant enhancement of reflectance in the same wavelength range. In particular, the Sb-codoped pigment exhibited the highest reflectance, which agreed well with its chromatic performance. For the W-codoped sample, a slight increase of reflectance could also be observed in the orange to red bands. However, the codoping of Mo even led to a much lower visible reflectance (18–21%) than only Cr-doping in the whole UV–Visible band, indicating that the Cr/Mo codoped TiO_2_ rutile sample of the pigment could absorb most of the UV–Visible light and thus appeared black. Figure 4a also shows that all the doped rutile pigments had relatively low reflectance to blue-green light, and the reflectance/absorption edge was red-shifted from ca. 500 to ca. 700 nm in the order of Sb, Nb, W, and Mo. The curves of the forbidden bandgaps derived from the spectra according to the Kubelka–Munk theory are shown in Figure 4b, where the values of the intersection of the tangent line and the horizontal axis are the values of the band gaps (E_g_). The obtained E_g_ values and corresponding intrinsic absorption wavelength λ_i_ (E_g_ = 1240/λ_i_) are listed in Table 2. It can be seen that the single Cr-doped pigment had an E_g_ of 1.83 eV. The doping of Sb, Nb, and W produced an increase in E_g_ to above 2.03 eV, while the introduction of Mo ions resulted in a significant reduction in E_g_ to 1.36 eV. These differences in bandgap can also be attributed to the different degrees of crystal structure distortion and defects caused by the cation doping. For the 10% Cr, Cr/Mo, and Cr/W doped TiO_2_, the generation of electronic defects due to charge imbalance creates additional energy levels in the band structure, which should be the main reason for the significant increase in visible light absorptance. The calculated values of E_g_ and λ_i_ well justify the color rendering characteristics of the as-synthesized pigments with different counterions.

**Figure 5:**
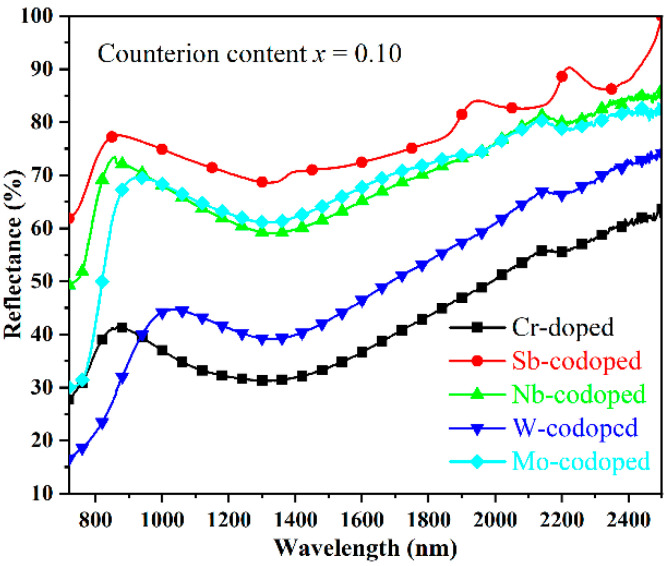
NIR reflectance spectra of the Cr-doped TiO_2_ pigments with and without counterions.

As is well-known, the solar spectrum consists of 48% of UV–Visible radiation and 52% of near-infrared radiation (NIR). The demand for near-infrared radiation heat-shielded pigments (so-called cool pigments) has been increasing in recent years due to the urban heat island (UHI) effect [41]. Therefore, the NIR reflectance properties from 780 to 2500 nm in wavelength of the doped TiO_2_ pigments were investigated, as shown in Figure 5. It was found that the codoping of Sb, Nb, and Mo resulted in a remarkable enhancement in the NIR reflectance of pigments, among which the Sb-codoped sample possessed the highest NIR reflectance of more than 69% in the whole NIR region. In contrast, the single Cr-doped sample showed relatively low NIR reflectance. With the W-codoping, a slight increase in the NIR reflectance in the wavelength of 940–2500 nm could also be observed. Furthermore, considering that the largest part of the NIR energy was distributed in the region with a wavelength below 900 nm, the rutile Ti_0.8_Cr_0.1_Sb_0.1_O_2_ and Ti_0.8_Cr_0.1_Nb_0.1_O_2_ pigments are preferred for energy-saving applications such as architectural coatings, vehicle paints, exterior wall tiles, and tile glazes.

**Figure 6:**
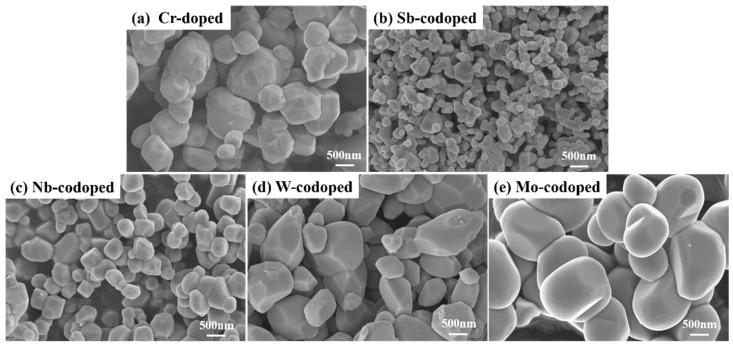
SEM images of the Cr-doped TiO_2_ pigments without (**a**) and with counterions: (**b**) Sb, (**c**) Nb, (**d**) W and (**e**) Mo.

Figure 6 illustrates the SEM images of the prepared Ti_0.9_Cr_0.1_O_2_ and Ti_0.8_Cr_0.1_M_0.1_O_2_ (M = Sb, Nb, W, Mo) pigments calcined at 1100 °C for 2 h. As seen in Figure 6a, the single Cr-doped pigment powder was composed of irregular and polyhedral grains with sizes mainly distributed from 500 to 1000 nm. Although the powders mostly maintained a characteristic polyhedral structure of the tetragonal crystal system at different degrees of integrity, their microscopic morphology changed significantly after the codoping of different counterions. As shown in Figure 6b, the 10% Sb-codoping contributed to grains with a significantly reduced size of 100–300 nm and good dispersion. A similar phenomenon could also be observed for the Nb-codoped powder, except that its particle size of 250–500 nm was a little larger than that of the Sb-codoped sample (Figure 6c). However, for the W and Mo codoped pigments, their maximum particle size was close to that of the single Cr-doped samples. As shown in the XRD pattern of Figure 2b, there should be a small amount of residual WO_3_ particles for the W-codoped samples in Figure 6d. However, due to the similar crystallization habits, it is difficult to distinguish the orthorhombic WO_3_ particles from the rutile particles in the SEM image. The grain growth behavior of the pigment powders during the heat-treatment process is understandable by considering the effect of doping such as the formation of defects and distortion of the crystalline lattice. The obtained results indicate that the surface diffusion barrier of grains was increased, and hence the grain growth was hindered with the addition of 10% Sb and Nb. It is also noteworthy that polyhedral particles with a smooth surface and relatively uniform size were formed when Mo was codoped (Figure 6e), which may be related to the generation of a little liquid phase during the powder synthesis process due to the relatively low melting point of MoO_3_. For the oxide pigments, the particle morphology and size of the powders have an important influence on their performance for various applications. For instance, when used as the ink pigments, the well-dispersed superfine particles derived from the Cr/Sb and Cr/Nb doping could help to shorten the grinding time and improve the fluidity of the ink, thereby improving the color performance and reducing the processing cost.

The synthesized Ti_0.9_Cr_0.1_O_2_ and Ti_0.8_Cr_0.1_M_0.1_O_2_ (M = Sb, Nb, W, and Mo) pigments were also treated with 5 wt% H_2_SO_4_, HCl, and NaOH aqueous solution for 60 min, respectively, in order to evaluate their chemical stability. No obvious mass loss was found for all the tested samples, suggesting that the obtained pigments had good resistance to acid and alkali corrosion. Table 3 presents the CIELab chromatic parameters of pigments before and after acid and alkali corrosion tests. For comparison, the calculated chromatic aberration indexes (ΔE*) were also included. It is evident that the chromaticity parameter L*, a*, b* values did not change much after the 5 wt% acid and alkali corrosion tests, and all the ΔE* values were less than 2.44, indicating that the synthesized pigments had excellent stability [42,43,44,45]. This is mainly due to the excellent chemical stability of fully crystallized rutile TiO_2_ subjected to high-temperature calcination. The doped Cr^3+^ and counterions were not easily leached out by dilute acid and alkali solutions after they solidified into the rutile lattice, which is beneficial in improving the stability of pigments in applications of paint and plastic coloring.

**Figure 7:**
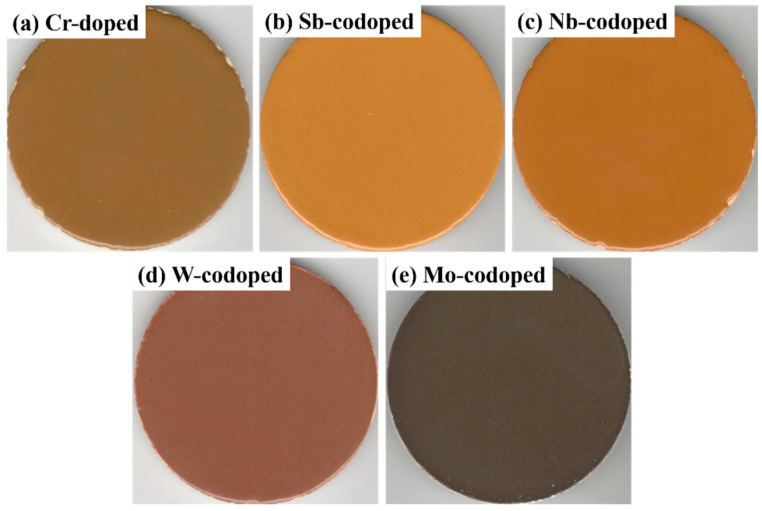
Photographs of the colored ceramic glazes with the Cr-doped TiO_2_ pigments without (**a**) and with counterions: (**b**) Sb, (**c**) Nb, (**d**) W, and (**e**) Mo.

It is well-known that the pigments applied for ceramic or enamel glaze coloring and glass decoration should have excellent color-rendering properties and high-temperature stability in the glaze or glass melts [46]. In this work, the prepared pigments (5% of the total weight of the glaze) were applied in a transparent glaze to investigate the coloring performance and hiding power as well as high-temperature chemical stability. Figure 7 shows the surface photos of the colored glazes prepared by sintering at 1000 °C for 20 min, and their corresponding colorimetric parameters are shown in Table 4. As can be seen, all the colored glazes exhibited sufficient hiding power to cover the ceramic bodies, which can be attributed to the strong opacifying ability of rutile TiO_2_ with high refractive index, and their color tones were similar to those of the respective pigments. In addition, the yellowness (b* value) was increased to some extent due to the synergistic effect of the glaze and pigment. These results show that the prepared pigments have good coloring power and stability in high-temperature glass fluxes, and have good application potential in ceramic, enamel, and glass decorations.

## 4. Conclusions

The rutile TiO_2_-based pigments codoped with chromophore ion of Cr^3+^ and various charge-balancing ions of Sb, Nb, W, and Mo were prepared by a low-cost solid-state reaction method. The introduction of counterions has significant effects on the phase composition, color-rendering, and spectroscopic properties. For the 10% Cr single-doped pigment, an obvious second-phase impurity of Ti_0.78_Cr_0.12_O_1.74_ was formed in addition to the rutile phase calcined at 1100 °C for 2 h. With the codoping of 5% and 10% counterion species (Sb, Nb, W, or Mo), all the obtained pigments possessed the main crystalline phase of the rutile structure, indicating that the addition of counterions effectively enhanced the solubility of Cr^3+^ in the TiO_2_ lattice. The pigment doped with only 10% Cr showed a dark brown color. With the codoping of 5% Sb, the yellow color was obtained, and much higher yellowness and chroma could be achieved with a further increase to 10%, while the addition of 10% Nb led to a yellowish-orange. The color of the rutile pigment can also be tailored to be reddish-orange and dark reddish-brown, respectively, at the W-doping level of 5% and 10%. However, both Mo-doped pigments appeared to have a similar black color. The influence of counterions on the chromatic performance of obtained rutile pigments could be attributed to the lattice distortion and variation in the charge balance condition depending on the radius and valence of codoped cations, which can change the crystal band structure and thus the visible light absorption/reflection properties. The addition of Sb, Nb, and Mo enhanced the NIR reflectance of pigments significantly in the whole NIR region. Furthermore, the inhibition of grain growth and better dispersion could also be achieved by the codoping of Sb or Nb, resulting in the nano-sized pigment powders. All the prepared rutile pigments exhibited good chemical stability subjected to the corrosion of dilute acid and alkali solutions as well as excellent color-rendering properties in the ceramic glaze. The obtained results show the effectiveness of counterions in tailoring the color-rendering performance of rutile pigments. Based on this, it can be predicted that the performance of rutile pigments with different hues can be further optimized by rational design of the chromophore ion–counterion pairs and their proportions. In the same way, the development of high NIR reflectance pigments as cool pigments is also worthy of research in the near future, especially in the wavelength of 750–1350 nm. Thus, the present research contributes to a good foundation for the development of eco-friendly pigments with the required color for diverse industrial applications.

## Figures and Tables

**Table 1:** Chemical composition of the transparent glaze powders.

Oxides	SiO_2_	Al_2_O_3_	CaO	BaO	MgO	Na_2_O	K_2_O	ZnO	ZrO_2_	Fe_2_O_3_
Content/%	65.70	13.00	5.84	4.00	0.86	3.52	0.79	0.51	5.50	0.20

**Table 2:** Intrinsic absorption bandgap (E_g_) and wavelength (λ_i_) of the doped TiO_2_ pigments.

Dopant Elements	Cr	Cr/Sb	Cr/Nb	Cr/W	Cr/Mo
E_g_/eV	1.83	2.28	2.21	2.03	1.36
λ_i_/nm	677	543	561	610	911

**Table 3:** Comparison of chromatic parameters for the Cr-doped TiO_2_ pigments with and without counterions before and after the acid and alkali corrosion tests.

Doped Elements	Conditions	L*	a*	b*	ΔE*
10% Cr	Untreated	41.06	12.00	17.54	-
	5% HCl	39.85	12.12	16.88	1.38
	5% H_2_SO_4_	40.25	12.64	17.85	1.08
	5% NaOH	39.70	12.60	19.44	2.41
10% Cr + 10% Sb	Untreated	62.75	25.15	46.64	-
	5% HCl	61.53	25.69	46.36	1.36
	5% H_2_SO_4_	61.82	25.69	46.02	1.24
	5% NaOH	61.55	25.26	46.23	1.27
10% Cr + 10% Nb	Untreated	52.13	26.14	41.44	-
	5% HCl	52.95	27.46	41.06	1.59
	5% H_2_SO_4_	52.20	27.98	41.18	1.86
	5% NaOH	52.67	28.04	41.52	1.98
10% Cr + 10% W	Untreated	36.44	21.68	13.69	-
	5% HCl	38.21	22.19	14.54	2.44
	5% H_2_SO_4_	38.65	21.84	13.96	2.23
	5% NaOH	36.78	23.03	14.21	1.49
10% Cr + 10% Mo	Untreated	29.17	3.60	−1.81	-
	5% HCl	28.11	2.79	−1.78	1.33
	5% H_2_SO_4_	28.63	2.74	−1.96	1.03
	5% NaOH	28.98	2.76	−2.61	1.18

ΔE* = [(ΔL*)^2^ + (Δa*)2 + (Δb*)^2^]^1/2^.

**Table 4:** Chromatic parameters of colored glazes with the doped TiO_2_ pigments.

Doped Elements	L*	a*	b*	C*
10% Cr	41.63	12.82	27.02	29.91
10% Cr + 10% Sb	54.28	21.96	49.46	54.12
10% Cr + 10% Nb	43.93	23.31	43.33	49.20
10% Cr + 10% W	37.15	16.38	14.21	21.68
10% Cr + 10% Mo	29.99	4.11	6.50	7.69

## Data Availability

Data is contained within the article.
